# Social determinants of mental health problems among South Asian migrants living in industrialized countries: a systematic review

**DOI:** 10.1093/pubmed/fdaf092

**Published:** 2025-08-07

**Authors:** Prashamsa Pandey, Saman Khalesi, Sophiya Dulal, Grish Paudel, Lal Rawal

**Affiliations:** School of Health, Medical and Applied Sciences, Central Queensland University, Sydney, 400 Kent Street, NSW 2000, Australia; Appleton Institute, Central Queensland University, Unit 8.2, 160 Ann Street, Brisbane, QLD 4000, Australia; School of Health Sciences, Western Sydney University, Locked Bag 1797, Penrith South DC, NSW 2751, Australia; School of Health, Medical and Applied Sciences, Central Queensland University, Sydney, 400 Kent Street, NSW 2000, Australia; School of Health, Medical and Applied Sciences, Central Queensland University, Sydney, 400 Kent Street, NSW 2000, Australia; Physical Activity Research Group, Appleton Institute, Central Queensland University, 554-700 Yaamba Road, Norman Gardens, Rockhampton 4700, Australia; Translational Health Research Institute (THRI), Western Sydney University, Locked Bag 1797, Penrith, NSW2751, Australia

**Keywords:** anxiety, depression, industrialized countries, South Asian migrants, stress

## Abstract

**Background:**

Migration involves a risk of mental health problems, including stress, anxiety, and depression. This study systematically reviewed social determinants of mental health problems among South Asian migrants living in industrialized countries.

**Methods:**

Four databases (PubMed, CINAHL, EMBASE, and PsycINFO) were searched for observational studies published between 2000 and 2025. The social ecological model was used as a theoretical framework. Studies that included adult South Asian migrants using validated mental health tools were included. Social determinants of mental health were identified through extraction of social factors that demonstrated statistically significant associations with mental health problems. The study protocol was registered in PROSPERO and followed PRISMA guidelines.

**Results:**

Seventeen studies met the inclusion criteria. The prevalence of stress, anxiety, and depression ranged from 23% to 59%, 20% to 50%, and 9% to 47%, respectively. Common social determinants of mental health problems were age, gender, marital status, social support, language, education, and employment. Being older, female, unmarried, or unemployed or having less social support or lower education, or facing language barriers were major factors influencing mental health problems.

**Conclusions:**

The findings warrant the development and implementation of policies focused on addressing these social determinants of mental health problems and improving access to and utilization of mental health services.

## Introduction

Migration is a common practice globally^1^^,^^2^. People migrate from low- and middle-income countries (LMICs) to high-income countries (HICs), in search of better education, employment opportunities, future security, and to escape from political persecution and marriage^1^^,^^3^^,^^4^. According to the International Organization for Migration (IOM), the number of international migrants increased from 153 million in 1990 to 281 million in 2020, of which 40% were born in Asia.^3^ Most South Asian migrants choose industrialized countries, including the USA, Canada, and Australia^5^.

While migration brings many opportunities, it poses challenges, including stress, strain, isolation, unemployment, and separation from family and relatives^2^^,^^6^. These factors can significantly impact physical, social, and mental health, leading to mental health problems such as stress, anxiety, and depression^6^^,^^7^. Studies suggest that South Asian migrants in industrialized countries experience a high burden of mental health problems compared to people in host countries^7–9^. Mental health problems are exacerbated by environmental changes, including discrimination, cultural conflicts, and the living standards experienced in host countries^10^^,^^11^. A study in Australia reported that one in six Indian Australians had a higher prevalence of depression compared to the general Australian population^12^. Similarly, studies among South Asian migrants in Hong Kong reported a prevalence of stress of 25%^13^ and in Greece, the prevalence of anxiety was 42%^14^. Further, in a systematic review of studies among South Asian postpartum mothers living in industrialized countries, a depression prevalence of 5%–20% was reported^15^.

The experience of mental health problems may differ due to social differences in different populations. Age, isolation, social support, gender, generation, employment, and language barriers are among these determinants^7^^,^^16^. Isolation, unemployment, and difficulties in coping with a new environment may result from poor language proficiency and social support^17^^,^^18^. Unemployment can increase difficulties in maintaining living expenses, which deteriorate the living standards of migrants^17^^,^^19^. Furthermore, the lack of communication with friends, family, and relatives can lead to isolation in host countries. Consequently, migrants feel disconnected from social networks in their home country and struggle to adapt and create new social networks in the host country^7^^,^^17^. Additionally, younger migrants were more likely to report mental health problems due to the challenges they face during migration and in adjusting to the new environment compared to other migrants^20^. Female migrants also often face challenges in balancing work, home responsibilities, childcare, academic development, and career planning. These factors contribute to a higher prevalence of mental health issues and a reduced capacity to cope, compared to their male counterparts^21^^,^^22^. Studies in the past have shown that South Asian migrants face a range of challenges, including cultural adjustment, language barriers, employment, and housing issues in settling down in industrialized countries^7^^,^^23^. These factors may contribute to the high levels of mental health problems that South Asian migrants experience in host industrialized countries^9^^,^^24^^,^^25^. However, the literature exploring these factors and their link to mental health problems in South Asian migrants is inconsistent. Variations in study designs, populations, and measurement tools have contributed to these inconsistencies in the findings. Therefore, this study systematically reviewed the published literature to determine the social determinants of mental health problems among South Asian migrants living in industrialized countries.

## Methods

This systematic review followed the Preferred Reporting Items for Systematic Reviews and Meta-Analysis (PRISMA) guidelines^26^. The South Asian countries included were Bangladesh, Bhutan, India, Nepal, Pakistan, Maldives, Sri Lanka, and Afghanistan^27^. The systematic review protocol was registered in the International Prospective Register of Systematic Reviews (PROSPERO) database (CRD42023477023).

### Search strategy

Four databases (PubMed, CINAHL, PsycINFO, and Embase) were searched for relevant literature published between January 2000 and June 2025, identifying a total of 9707 articles. The key search terms included ‘stress’, ‘anxiety’, ‘depression’, ‘South Asia’, and ‘industrialized countries’^28^. Medical Subject Headings (MeSH), Boolean operators, wild cards, and field tags were used (see Supplementary Table 1 for key search terms). Necessary support from the Central Queensland University librarian was sought to finalize search terms and strategies to ensure a quality search and minimize the risk of missing relevant studies.

### Inclusion and exclusion criteria

A summary of the inclusion and exclusion criteria is presented in Table 1. This review included observational quantitative studies that focused on mental health problems such as stress, anxiety, and depression among adult South Asian migrants living in industrialized countries. The eligible studies used validated self-reported assessment tools with demonstrated validity and reliability in the literature, including the Depression Anxiety and Stress Scale-21^29^, Kessler Psychological Distress Scale^30^, Center for Epidemiologic Studies Depression Scale^31^, Acculturative Stress Scale^32^, and General Health Questionnaire^33^, among South Asian adult migrants living in industrialized countries. Studies among refugees and asylum seekers or participants with severe mental health problems, including major depression and schizophrenia, were excluded to limit the influence of these conditions or medical treatment on the associations between social determinants and mental health outcomes.

**Table 1:** Study selection criteria.

Parameters	Inclusion criteria	Exclusion criteria
Setting	Industrialized country/High-income country	Low- and middle-income countries
Population	18+ years, South Asians, living in industrialized countries	Literature that does not focus on mental health (stress, anxiety, and depression) and asylum seekers
Phenomena	Mental health problems include stress, anxiety, and depression, which have been used with valid tools such as the Depression Anxiety and Stress Scale (DASS21) or Kessler Psychological Distress Scale (K6), Center for Epidemiologic Studies Depression Scale (CES-D), Acculturative Stress Scale, General Health Questionnaire, Psychological Well-being Questionnaire, Edinburgh Postnatal Depression Scale (EPDS), Geriatric Depression Scale (GDS), General Anxiety Disorder-7 (GAD-7), Social Attitudinal Familial Environmental Stress Scale, COVID-19 distress scale, the Zung Self-Rating Depression Scale, and chronic stress: Index of residential crowding demand/control financial strain neighbourhood stress	Those patients that are receiving medical treatment or interventions for stress, anxiety, and depression or are diagnosed with other more severe mental health problems (e.g. major depression, schizophrenia)
Study design	Quantitative research: cross-sectional study	Published literature other than quantitative research
Social determinants	Any of the social determinants of stress, anxiety, and depression should be included in the study	Studies without any association between mental health problems and its social determinants
Publication date	1 January 2000 to January 2025	Before 1 January 2000
Language	Published in English	Other languages

### Study screening and data extraction

The literature identified from the initial search was imported into EndNote 20^34^. After removing duplicates, two reviewers (P.P. and G.P.) screened titles and abstracts independently. Any disagreements were resolved by involving the third and fourth reviewers (L.R. and S.K.). Forward and backward reference searches were conducted for the articles to identify additional studies. Data was extracted using a modified version of the data extraction tool developed by Nafisa Insan *et al.* (2022)^35^. Information on primary author, publication year, country, study design, sample size, participants’ country, outcome measures, and social determinants of stress, anxiety, and depression was extracted.

### Quality appraisal

The Newcastle Ottawa Scale (NOS)^36^ was used to assess the quality of the cross-sectional studies included^36^ by two independent reviewers (P.P. and G.P.). The scale included three dimensions: selection, comparability, and exposure. The maximum score possible for a given study was 10 points. Studies were categorized as follows: those scoring 0–4 points, 5–6 points, 7–8 points, or 9–10 points were considered ‘unsatisfactory’, ‘satisfactory’, ‘good’, or ‘very good’, respectively Table 2).^37^

**Table 2:** Quality assessment of the studies included in the review^a^.

Author, year	Selection			Comparability		Outcome		Total score (10)^b^
	Representativeness of the sample	Sample size	Nonrespondents	Ascertainment of the exposure	Control of confounding factors	Assessment of the outcome	Statistical test	
	Score (1)	Score (1)	Score (1)	Score (2)	Score (1)	Score (2)	Score (2)	
*India (*n* = 4)*								
Diwan, 2008	1	1	1	2	1	1	1	8
Atri et al., 2006	1	1	0	2	1	1	1	7
Kateri et al., 2019	1	1	0	1	1	2	1	7
Goyal, 2005	1	0	0	1	1	2	1	6
*Pakistan (*n* = 1)*								
Jibeen and Khalid, 2010	1	1	0	2	1	2	1	8
*Nepal (*n* = 1)*								
Khatiwada et al, 2021	1	1	0	2	1	2	1	8
*South Asia (*n* = 11)*								
Dhillon and Macarthur, 2012	1	1	0	2	1	1	1	7
Aujla *et al.*, 2010	1	1	1	2	1	1	1	8
Wong *et al.*, 2022	1	1	1	2	1	1	1	8
Lai *et al.*, 2008	1	1	0	2	1	1	1	7
Roberts *et al.*, 2015	1	1	0	2	1	1	2	8
Lozano *et al.*, 2022	1	1	1	2	1	1	1	8
Tonsing *et al.*, 2016	1	1	0	2	1	2	1	8
Almeida *et al.*, 2023	1	1	1	1	1	2	1	8
Abouguendia *et al.*, 2001	1	1	1	2	1	1	1	8
Grace *et al.*, 2016	1	1	0	1	1	2	1	7
Williams *et al.*, 2007	1	0	0	1	1	2	1	6

^a^Very good studies: 9–10 points; good studies: 7–8 points; satisfactory studies: 5–6 points; unsatisfactory studies: 0–4 points.

^b^Newcastle–Ottawa scale.

### Social–ecological approach to understanding the social determinants of mental health

**Figure 1:**
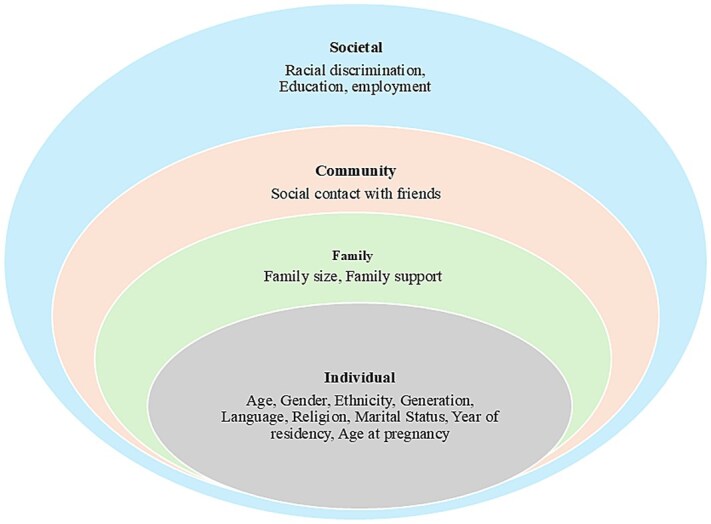
The ecological model adapted for social determinants of mental health problems at the individual, family, community, and societal levels that influence mental health problems adapted from Bronfenbrenner (1979).

The social ecological model (SEM) has been used broadly, including efforts to understand health and well-being, ranging from individual to societal levels^38–40^. In this systematic review, the SEM^41^ was used to identify and understand the influence of social determinants on mental health problems among South Asian migrants living in industrialized countries. Therefore, the SEM framework guided this study to identify social determinants of mental health problems, as shown in Figure 1.

## Results

### Search results

**Figure 2:**
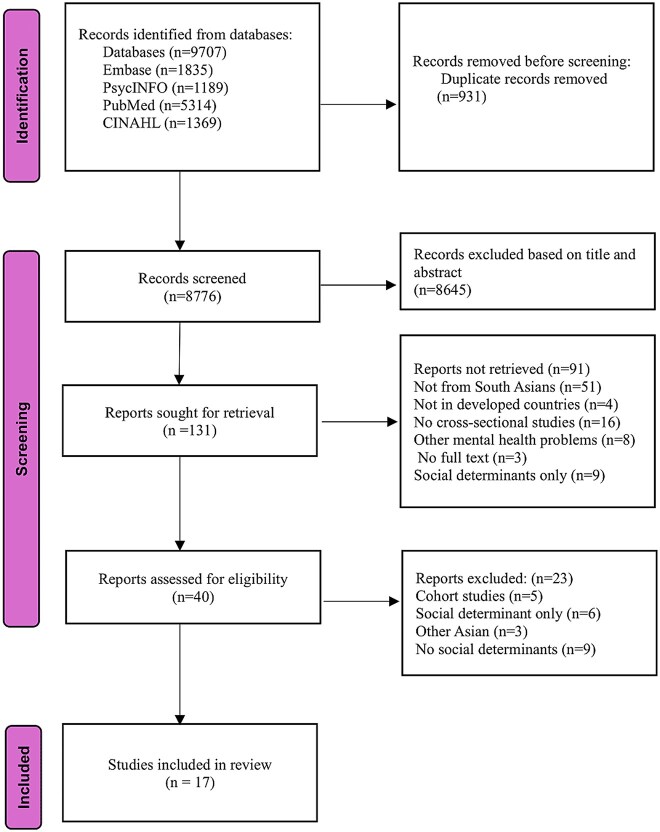
Preferred Reporting Items for Systematic Reviews and Meta-analysis flow diagram describing the process of article selection.

A total of 9306 studies were identified through the initial search of the literature. After removing duplicates (*n* = 909), 8397 studies were screened for titles and abstracts. Of these, 8267 studies were excluded, and 130 were eligible for full-text review. After full-text review, 17 studies met the inclusion criteria and were included. Figure 2 presents the PRISMA flow diagram illustrating the article selection, inclusion, and exclusion process.

### Risk of bias assessment

An overview of the quality assessment of the studies, conducted using NOS^36^, ranged from very good to unsatisfactory, as shown in Table 3. In total, 15 studies (88%) were classified as good, and 2 (12%) were classified as satisfactory. Over half (58%) of the studies did not discuss nonrespondents adequately. Two studies did not meet the sample size criteria, as they had fewer than 100 participants^42^^,^^43^. For the outcome component, 76% of the studies had nominal descriptions and inadequate discussion on the measurement of association, confidence intervals, and probability levels (*P*-values).

**Table 3:** General characteristics of the studies included in this review.

Author, year	Study design/Sample size	Residing industrialized country	Sample characteristics	Determinants	Outcome measures
*India origin migrants (*n* = 4)*					
Diwan, 2008	A cross-sectional study. Sample 1: 263 (English-speaking Indians) Sample 2: 114 (non-English-speaking Indians)	USA	First-generation Indians Male: sample 1—199; sample 2—54 Female: sample 1—64; sample 2—60 The mean age of sample 1 was 57.6 and that of sample 2 was 61.4 years	Education Gender Age Marital status Living arrangement Social contact with friends	Use of Center for Epidemiologic Studies Depression Scale (CES-D). A total of 7% of participants in sample 1 and 11% in sample 2 had some form of depression For women, the rate of increased depressive symptoms was 120% greater compared to men )
Atri *et al.*, 2006	A cross-sectional study Total sample: 185 Indians	USA	Mixed-generation Indians Male: 114 Female: 71 The mean age of Indians was 26.15 years	Age Length of stay Gender Education Occupation Language Social support	Use of Kessler Psychological Distress Scale (K-6)
Kateri *et al.*, 2019	A cross-sectional study Total sample: 114 Indian adults	Greece	First-generation Indians Male: 85 Female: 29 The mean age was 51.3 years	Age Education Years of residency Social support	Use of CES-D State Anxiety Inventory The average anxiety score (State Anxiety) for the sample was 41.5 The average depression score (CES-D) for the sample was 17.4
Goyal, 2005	A cross-sectional study Total sample: 58 Indians	USA	Mixed-generation Indians Female: 58 The mean age of Indians was 29 years	Age Length of stay Married Education Arranged marriage Gender of infant	Postpartum Depression Screening Scale (PPDS) Minor depressive symptomatology rate of 28% and an additional major depressive symptomatology rate of 24%
*Pakistan origin migrants (*n* = 1)*					
Jibeen and Khalid, 2010	A cross-sectional study Total sample: 214 Pakistanis	Canada	First-generation Male: 113 Female: 101 The mean age of Pakistanis was 35.79 years	Income Education Occupation Age Gender Employment Length of residency, generation	Acculturative Stress Scale General Health Questionnaire (GHQ) Psychological Well-being Questionnaire The mean of stress was 12.35 (SD: 5,67), and GHQ was 13.92.
*Nepalese-origin migrants (*n* = 1)*					
Khatiwada, 2021	A cross-sectional study. Total sample: 249 Nepalese	Japan	Mixed generation Male: 166 Female: 83 The mean age of Nepalese individuals was 31.8 years	Age Marital status Ethnicity Education Language Length of stay Occupation Social support Satisfaction with life; in particular, the family, friends Satisfaction with life	GHQ
*South Asian countries origin migrants (*n* = 11)*					
Dhillon and Macarthur, 2012	Cross-sectional study in antenatal setting South Asian female: 300	UK	Mixed generation from India, Pakistan, and Bangladesh with a mean age of 28.6 years	Age Ethnicity Country of birth Education/married Employed Type of family A number of people providing support Quality of support from family and friends as rated by women Support from husband	Edinburgh Postnatal Depression Scale (EPDS) The prevalence of any form of depression was 30.70%
Aujla *et al.*, 2010	A cross-sectional study in the hospital South Asians: 290 White Europeans: 864	UK	Mixed generation South Asian migrants: male—143 Female- 147 The mean age of South Asian migrants was 51.3 years old	Age Sex Ethnicity	CES-D AND WHO-5 CES-D with type 2 DM 31.8% and Impaired Glucose Regulation (IGR) 40.7% WHO-5 with type 2 DM 28.6% and IGR 45.8%
Wong *et al.*, 2022	A cross-sectional study 310 South Asians	Hong Kong	Mixed generation Pakistan: 136; India: 100; Nepal: 69; others: 5 Male-119 Female- 191 The mean age of South Asian migrants was 41.3 years old	Age Gender Place of birth Country of origin Income Work status Occupation Marital status Religion Knowledge about COVID-19 Worry about losing a job	Depression Anxiety and Stress Scale-21 (DASS-21) Stress: Mean (SD): 18.4 (6.1); normal (0–14): 155 (50.0); mild (15–18): 50 (16.1); moderate (19–25): 53 (17.1); severe (26–33): 42 (13.5); extremely severe (≥34):10 (3.2) Anxiety: Mean (SD):17.8 (5.5); moderate (10–24): 162 (52.3); severe (15–19): 50 (16.1); extremely severe (≥20): 98 (31.6) Depression: Mean (SD): 18.5 (6.0); moderate (14–20): 225 (72.6); severe (21–27): 40 (12.9); extremely severe (≥28): 45 (14.5)
Lai *et al.*, 2008	Cross-sectional study 220 South Asians	Canada	First-generation South Asians include people from Bangladesh, India, Pakistan, Sri Lanka, and Nepal Male: 122 Female: 98 The mean age of South Asian migrants was 65.8 years	Age Gender Married Income Education Length of residency Social support Language Cultural values	Geriatric Depression Scale (GDS), 15 items The prevalence of depression was 21.40%
Roberts *et al.*, 2015	Mixed-methods research 350 for a quantitative survey and 58 for a qualitative study	USA	Mixed generation from India and others (46 were US-born, 293 were Indian-born, and 9 were others) Male: 133 (quantitative) and 22 (qualitative) Female: 127 (quantitative) and 36 (qualitative) The mean age of South Asian migrants was 41.85 years	Marital status Age Education Employment Ideal family size Religious coping Age at first pregnancy Domestic violence myth acceptance	Patient Health Questionnaire 9 (PHQ-9) for depression and General Anxiety Disorder-7 (GAD-7) for anxiety The prevalence of anxiety was mean 5.49 (SD: 5.27) and depression mean 5.04 (SD:5.32)
Lozano *et al.*, 2022	Cross-sectional study 636 [387 were from China (150 pre-COVID and 237 were during COVID) and 249 were from South Asia (138 pre-COVID and 111 during COVID); 280 were male and 356 were female)]	USA	Mixed generation from South Asia (India, Pakistan, Bangladesh, Sri Lanka, Afghanistan, and Nepal) Male: 117 South Asian (62 pre-COVID and 55 during COVID) Female: 132 South Asian (76 pre-COVID and 56 during COVID)	Age Education Employment Length of residency, generation Gender	Patient Health Questionnaire Eight-Item Depression Scale (PHQ-8) The prevalence of depression before COVID-19 was 8.68% and after COVID-19 was 20.68% Depression symptoms increased more than two-fold, from 9% pre-pandemic to 21% during the COVID-19 pandemic
Tonsing *et al.*, 2016	A cross-sectional study in the community. 218 Nepalese, 229 Pakistan	Hongkong	Mixed generation from Pakistan and Nepal Pakistan- male = 103, female-126 Nepal- male = 106, female- 112 The mean age was 30.6 years old.	Gender Employment, Marital status Education, Income, Age, Length of stay,	Depression Anxiety Stress Scale (DASS-21) Social Attitudinal Familial Environmental Stress Scale The prevalence was Stress: Mild: Pakistani—10.5%, Nepalese—11.0% Moderate: Pakistani—11.8%; Nepalese—10.6% Severe: Pakistani—8.3%; Nepalese—2.8% Extremely severe: Pakistani—0.9%; Nepalese—0.9% Moderate and above: Pakistani—21.0%; Nepalese—14.3% Anxiety: Mild: Pakistani—10.5%; Nepalese—6.9% Moderate: Pakistani—13.1%; Nepalese:—13.3% Severe: Pakistani—5.7%; Nepalese—2.8% Extremely severe: Pakistani—0.4%; Nepalese—0.5% Moderate and above: Pakistani—19.2%; Nepalese—16.6% Depression: Mild: Pakistani—14.0%; Nepalese—12.8% Moderate: Pakistani—10.5%; Nepalese—7.8% Severe: Pakistani—3.5%; Nepalese—3.7% Extremely severe: Pakistani—1.3%; Nepalese—0.5% Moderate and above: Pakistani—15.3%; Nepalese—12.0%
Almeida *et al.*, 2023	A cross-sectional study Total sample: 196	USA	Mixed generation from India, Pakistan, Bangladesh, Sri Lanka, or Nepal Male: 106 Female: 89 The mean age of South Asians was 34.51 years	Generation Age Place of birth Gender	COVID-19 distress scale The prevalence of stress was 22.88%
Abouguendia *et al.*, 2001	A cross-sectional study Total: 74; First-generation (G1): 40, Second generation (G2): 34	Canada	Mixed generation from South Asians G1 males—25 (62.5%) and G2 males —10 (29.4) Total: 40 G1 females—15 (37.5%) and G2 females—24 (70.6%); Total: 34 The mean age of G1 was 20.82 years and that of G2 was 19.68 years	Age Gender Generation Language Family support Place of birth Length of stay	The Zung Self-Rating Depression Scale (SDS; Zung, 1965)
Grace *et al.*, 2016	A cross-sectional study Total sample: 225	Canada	Mixed generation from India, Sri Lanka, Pakistan, and Bangladesh Female: 81 The mean age of South Asian migrants was 55.64 ± 8.89 years	Gender Education Language Work status Income Own home Perceived health	Epidemiological Studies Depressive Symptoms Scale (CES-D) Generalized Anxiety Disorder Scale (GAD-7) The prevalence of anxiety was Mild: 1022 participants (13.6%) Moderate: 324 participants (4.3%) Severe: 153 participants (2.0%) The prevalence of depression was 46.40%
Williams *et al.*, 2007	A cross-sectional study 63 South Asians and 42 white Europeans aged 35–75 years	UK	First generation from India (58.7%), Pakistan (9.5%), Bangladesh, and Sri Lanka Male- 63 The mean age of South Asian migrants was 51.3 ± 12.5 years	Age Married Employment Racial harassment Community support Lower emotional support	Center for Epidemiologic Studies of Depression Scale (CES-D) Chronic stress: Index of residential crowding demand/control model financial strain neighbourhood stress The prevalence of anxiety was 51.4% and depression was 20.8%

### Population characteristics

Study characteristics are presented in Table 2. Six of seventeen studies were conducted in the USA^20^^,^^24^^,^^43–46^, four in Canada^47–50^, three in the UK^42^^,^^51^^,^^52^, two in Hong Kong^9^^,^^13^, one in Japan^25^, and one in Greece^14^. The participants included in these studies were from India (4), Pakistan (1), Nepal (1), and South Asian countries (11). Participants’ ages ranged from 19 to 65 years. The total number of participants was 3636 (1728 males and 1908 females).

### Prevalence of stress, anxiety, and depression

The prevalence of stress, anxiety, and depression is presented in Table 3. The prevalence of stress was 23%–59%^23^^,^^42^^,^^46^, anxiety was 20%–51%^42^^,^^50^, and depression was 9%–47%^44^^,^^50^.

### Social determinants of stress, anxiety, and depression

Social determinants of stress, anxiety, and depression according to the SEM are presented in Figure 1 and Table 4. Factors at the individual level were personal characteristics, including age, gender, ethnicity, language, religion, marital status, economic status, and year of residency. Two studies showed that age influences an individual’s ability to cope with stress, adapt to life changes, and manage mental and physical health^14^^,^^20^. Four studies reported gender as a significant influencer of mental health^20^^,^^44^^,^^48^^,^^50^. These studies reported that women are more likely to experience higher levels of depressive symptoms in South Asian migrants living in industrialized countries^24^^,^^50^^,^^51^. Two studies found that individuals who are single, divorced, or widowed are likely to experience higher levels of depression and stress^9^^,^^23^. Similarly, one study highlighted that language barriers, religion, and ethnicity can lead to social isolation, misunderstandings, and reduced access to services^50^. Also, one study reported that the length of time an individual has lived in a particular country can influence their mental health and suggested that newer migrants may experience higher levels of stress and mental health issues due to acculturation challenges, language barriers, and lack of social support^20^.

**Table 4:** Association between social determinants and mental health problems among south Asian migrants living in industrialized countries.

Author, year	Social determinants of mental health problems														
	Age	Gender	Ethnicity	Education	Generation	Language	Family size	Religion	Marital status	Employed/Income	Year of residency	Racial harassment/Discrimination	Social contact with friends and family	Age at first pregnancy	Outcomes
*India (*n* = 4)*															
Diwan, 2008		^**^		^**^											Center for Epidemiologic Studies Depression scale (CES-D).
Atri *et al.*, 2006															Kessler Psychological Distress Scale (K-6)
Kateri *et al.*, 2019	^*^			^*^									^*^		CES-D State Anxiety Inventory
Goyal, 2005															Postpartum Depression Screening Scale (PPDS)
*Pakistan (*n* = 1)*															
Jibeen and Khalid, 2010			^**^									^**^			Acculturative Stress Scale General Health Questionnaire Psychological Well-being Questionnaire
*Nepal (*n* = 1)*															
Khatiwada *et al.*, 2021													^**^		General Health Questionnaire
*South Asia (n = 11)*															
Dhillon and Macarthur, 2012															Edinburgh Postnatal Depression Scale (EPDS)
Aujla *et al.*, 2010															CES-D and WHO-5
Wong *et al.*, 2022								^**^	^*^	^**^					Depression Anxiety and Stress Scale-21 (DASS-21)
Lai *et al.*, 2008		^**^													15-item Geriatric Depression Scale (GDS)
Roberts *et al.*, 2015							^**^							^*^	Patient Health Questionnaire 9 (PHQ-9) for depression and General Anxiety Disorder-7 (GAD-7) for anxiety
Lozano *et al.*, 2022	^***^	^*^								^*^	^*^				Patient Health Questionnaire eight-item depression scale (PHQ-8)
Tonsing *et al.*, 2016															Depression Anxiety Stress Scale (DASS-21) Social Attitudinal Familial Environmental Stress Scale
Almeida *et al.*, 2023															COVID-19 distress scale
Abouguendia *et al.*, 2001													^*^		The Zung Self-Rating Depression Scale (SDS; Zung, 1965)
Grace *et al.*, 2016		^***^				^***^			^***^	^***^					Epidemiological Studies Depressive Symptoms Scale (CES-D) Generalized Anxiety Disorder Scale (GAD-7)
Emily D. Williams *et al.*, 2007									^***^	^*^		^*^	^*^		CES-D Chronic stress: Index of Residential Crowding Demand/Control model Financial strain Neighbourhood stress

Note:

^*^ *P* < .05.

^**^ *P* < .01.

^***^ *P* < .001.

At the family level, the most important factor was family support and influence. Four studies found a significant positive association between family support and mental health^24^^,^^25^^,^^45^^,^^51^. Four studies highlighted that both the presence and quality of support from family, friends, and significant others are crucial for managing stress and improving mental health^24^^,^^25^^,^^45^^,^^51^. At the community level, engagement with friends and families, social connection with colleagues, and comparison of day-to-day activities and living standards influence the risk of mental health problems^24^^,^^25^. Four studies reported that religion, language, and ethnicity may influence social contact in the community^14^^,^^25^^,^^42^^,^^49^. The combination of reduced emotional support and diminished neighbourhood cohesion contributes to elevated stress and depression levels among South Asians in industrialized countries^42^.

At the societal level, two studies discussed racial discrimination among South Asians in industrialized countries^42^^,^^47^. Research indicates that racial and ethnic discrimination is a significant factor contributing to mental health challenges faced by migrants^47^. Education, employment, and income are significantly associated with the mental health problems of South Asian migrants in industrialized countries^14^^,^^20^^,^^42^^,^^44^^,^^50^. Changes in employment and income during migration can influence mental health, making it more challenging to maintain living standards and cope with the related difficulties^42^^,^^50^.

## Discussion

### Main findings of the study

This systematic review identified the prevalence and social determinants of mental health problems, as well as general characteristics among South Asian migrants living in industrialized countries. Further, using the SEM as a guiding theoretical framework, this review examined the association between mental health problems and social determinants of mental health among these populations at the individual, family, community, and societal levels. The findings of this review suggested a high prevalence of mental health problems among migrants from LMICs, particularly among those from South Asian regions^4^^,^^53–55^. These findings corroborate the findings of other studies conducted among migrants from other regions. For instance, a study conducted in the Netherlands found that the prevalence of depression was 33.6% among Moroccan elderly migrants and 61.5% among Turkish elderly migrants in 2004^54^. Similarly, a systematic review among African migrants in 2022 reported high prevalence rates of anxiety and depression, at 34.60% and 33.20%, respectively^53^. These findings underscore that migrants from LMICs to industrialized countries often experience mental health problems.

Most of the studies identified age as a common determinant of mental health problems. A systematic review among immigrants and refugees in 2021 indicated that age is a significant factor among migrants and refugees living in industrialized countries^56^. Overall, the review highlighted that younger migrants have a higher prevalence of mental health problems due to adaptation-related challenges.

Most of the studies reported gender as a key social determinant of mental health problems. This is supported by two studies—one conducted on Turkish migrants in Australia in 2007 and another on Nigerian migrants in Canada in 2023, which reported a higher prevalence of mental health problems, particularly depression, among females than among males^57^^,^^58^. However, a study among African migrants in the USA in 2020 did not show significant differences in anxiety and depression between genders^59^. In conclusion, female migrants may be at higher risk of mental health problems; further research is needed to explore gender differences in mental health problems, as existing studies present inconsistent findings.

This review found that marital status is a significant factor contributing to mental health problems among South Asian migrants^9^^,^^42^^,^^50^. The findings corroborate those of other studies. For instance, a study among African asylum seekers and refugees in Hong Kong in 2020 found that marital status significantly influences mental health, with married individuals reporting better mental health than those living alone^60^. Likewise, a study in the USA among Haitians found marital status as a strong predictor of depression^61^. Several studies have emphasized that spousal support is crucial during migration^9^^,^^42^^,^^50^. Two studies identified language as another major factor influencing mental health problems^44^^,^^50^. One study suggested the significance of language barriers in racial discrimination and cultural differences, which contribute to mental health problems^62^. The findings underscore the importance of addressing language barriers in mental health assessments and interventions, as individuals with limited proficiency may be at increased risk of mental health problems.

Four studies found that support from friends and family significantly reduced the prevalence of mental health problems^24^^,^^25^^,^^45^^,^^51^. However, one study among Indian migrants in the USA in 2008 found no association between social support and mental health, particularly for those with limited English-speaking skills, as they felt satisfied with their existing social networks^44^. The findings underscore the critical need for social support among South Asian migrants to promote their mental health.

Two studies indicated a positive association between education and mental health problems^14^^,^^44^. Evidence suggests that lower educational levels among Haitian migrants in the USA in 2020 are strongly associated with a higher incidence of mental disorders, including severe depression^61^. Four of eleven studies found that employment is crucial for ensuring stability, maintaining living standards, and increasing social interactions^9^^,^^20^^,^^42^^,^^50^. A study among Korean immigrants in the USA in 2011 stated that employment plays a crucial role in improving mental health by mitigating increased stress and challenges faced by individuals in lower economic classes^63^. This highlights the critical importance of educational and employment opportunities as key factors in addressing mental health challenges and enhancing overall well-being.

### What is already known on this topic

Studies in the past have highlighted that migrants from South Asian countries face higher levels of mental health problems when settling in new environments^6^^,^^44^^,^^48^ and the findings presented by these studies vary. Factors such as age, gender, education, and employment have been identified as contributors to mental health challenges in these populations. To date, no study has systematically reviewed and assessed the social determinants of the mental health problems faced by South Asian migrants living in industrialized countries.

### What this study adds

This systematic review highlights key factors such as age, gender, social support, education, employment, and language as significant influences on mental health. This review suggested that stress, anxiety, and depression are prevalent mental health problems among migrants from South Asian countries, with a high prevalence associated with settling in new environments. The findings highlighted the urgent need for developing policies and programmes that address the social determinants of mental health problems and improve mental health services, emphasizing the importance of targeted interventions among South Asian migrants living in industrialized countries.

### Limitations

This review has several important limitations. It primarily focused on quantitative studies and did not include qualitative studies, which could have deeper exploration of the determinants. Additionally, no studies were found on migrants from Afghanistan, Bhutan, and Maldives living in industrialized countries, which may limit the social determinants of mental health problems reported by this study. Also, studies that did not differentiate between Asian populations were excluded, which reduced the number of included studies. The studies used various scales to measure stress, anxiety, and depression, making it difficult to compare the results. Furthermore, due to heterogeneity in the scales used, data collection methods, and variables, a meta-analysis could not be conducted, which limited the interpretation of the findings. Lastly, while the SEM highlights policy as an important factor, this review did not focus on it, as most studies provided recommendations without discussing specific policies in detail.

## Conclusion

The findings of studies included in this review indicate a higher prevalence of mental health problems among South Asian migrants living in industrialized countries. Various social determinants at the individual, family, community, and societal levels contribute to the development of mental health problems among South Asian migrants. These include age, gender, marital status, social support, language, education, and employment. To address the mental health problems faced by South Asian migrants, it is crucial to design and implement culturally appropriate, cost-effective interventions targeted at migrant populations. Additionally, younger people, women, and those with lower education and limited language skills evidenced higher mental health problems. Future policies and programmes should focus on raising awareness and improving the utilization of mental health services among South Asian migrants. This can be done by promoting social integration, the availability of culture-friendly trained health professionals, and easy access to and use of health education materials and addressing barriers to care.

## Supplementary Material

Supplementary_material_table_1_fdaf092

## Data Availability

All data are presented in this systematic review, and no additional data are available.

## Notes

**Prashamsa Pandey,** Master's of Research Student/Researcher

**Saman Khalesi,** Senior Lecturer

**Sophiya Dulal,** PhD, Researcher

**Grish Paudel,** PhD Scholar, Researcher

**Lal Rawal,** Senior Lecturer
