# Supplementary material for: Social determinants of mental health problems among South Asian migrants living in industrialized countries: a systematic review
Source: J Public Health (Oxf). 2025 Aug 7;47(4):e652–67. doi: 10.1093/pubmed/fdaf092 (PMC12669993; doi:10.1093/pubmed/fdaf092)
Supplement: Supplementary_material_table_1_fdaf092 [file supplementary_material_table_1_fdaf092.docx]

Supplementary Table 1: Search terms

| **Database** | **Search** |
| --- | --- |
| EMBASE | 'depression'/exp OR 'anxiety disorder'/exp OR Stress OR Depression OR Anxiety OR “acculturative stress” OR discrimination OR “racial discrimination” OR “social support” AND 'developed country'/exp OR “industrialised countr*” OR “industrialized countr*” “high-income countr*” AND factor* OR “social determinant*” AND Immigrant* OR “CALD population*” OR Afghanistan OR Pakistan OR Nepal OR Bangladesh OR Bhutan OR India OR Maldives OR “Sri Lanka” OR “first-generation” OR 'migrant'/syn OR 'migration'/syn OR 'South Asia'/syn OR Romania OR Panama OR Chile OR “Trinidad and Tobago” OR Uruguay OR Antigua and Barbuda OR Barbados OR Poland OR Hungary OR Croatia OR Oman OR Nauru OR Curacao OR “Saint Kitts” and Nevis OR “French Polynesia” OR Latvia OR Greece OR Slovakia OR Lithuania OR “Puerto Rica” OR “Turks and Caicos Islands” OR “Saudi Arabia” OR “Czech Republic” OR Bahrain OR Estonia OR Bahamas OR Slovenia OR Cyprus OR Aruba OR Brunei OR Malta OR “Sint Maarten” OR Greenland OR Kuwait OR “South Korea” OR Italy OR “New Caledonia” OR UAE OR Andorra OR Macau OR “San Marino” OR Israel OR Belgium OR “Hong Kong” OR Sweden OR “Cayman Islands” OR Qatar OR Singapore OR Iceland OR “Faroe Islands” OR Norway OR Luxembourg OR “Isle of Man” OR Ireland OR Bermunda OR USA OR UK OR Canada OR Switzerland OR Germany OR France OR Australia OR Netherlands OR “New Zealand” OR Finland OR Japan OR Denmark |
| PubMed | Migrant* OR Immigrant* OR Migration OR CALD population* OR “South Asia*” OR Afghanistan OR Pakistan OR Nepal OR Bangladesh OR Bhutan OR India OR Maldives OR “Sri Lanka” OR “first-generation” OR “Transients and Migrants"[Mesh] OR "Emigrants and Immigrants"[Mesh] OR "Emigration and Immigration"[Mesh] OR "South Asian People"[Mesh] OR "Asia, Southern"[Mesh] AND  “industrialised countr*” OR “industrialized countr*” OR “high-income countr*” OR “developed countr*” OR "Developed Countries"[Mesh] OR Romania OR Panama OR Chile OR Trinidad and Tobago OR Uruguay OR Antigua and Barbuda OR Barbados OR Poland OR Hungary OR Croatia OR Oman OR Nauru OR Curacao OR “Saint Kitts” and Nevis OR “French Polynesia” OR Latvia OR Greece OR Slovakia OR Lithuania OR “Puerto Rica” OR “Turks and Caicos Islands” OR “Saudi Arabia” OR “Czech Republic” OR Bahrain OR Estonia OR Bahamas OR Slovenia OR Cyprus OR Aruba OR Brunei OR Malta OR “Sint Maarten” OR Greenland OR Kuwait OR “South Korea” OR Italy OR “New Caledonia” OR UAE OR Andorra OR Macau OR “San Marino” OR Israel OR Belgium OR “Hong Kong” OR Sweden OR “Cayman Islands” OR Qatar OR Singapore OR Iceland OR “Faroe Islands” OR Norway OR Luxembourg OR “Isle of Man” OR Ireland OR Bermunda OR USA OR UK OR Canada OR Switzerland OR Germany OR France OR Australia OR Netherlands OR “New Zealand” OR Finland OR Japan OR Denmark  AND  factor* OR “social determinant*” OR "Sociodemographic Factors"[Mesh] OR "Social Factors"[Mesh] OR "Environmental Biomarkers"[Mesh] AND  Stress OR Depression OR Anxiety OR “acculturative stress” OR discrimination OR “racial discrimination” OR “social support” OR "Mental Health"[Mesh] OR "Mental Disorders"[Mesh] OR "Anxiety Disorders"[Mesh] OR "Depressive Disorder"[Mesh] |
| CINAHYL | Migrant* OR Immigrant* OR Migration OR CALD population* OR “South Asia*” OR “South Asian” OR Afghanistan OR Pakistan OR Nepal OR Bangladesh OR Bhutan OR India OR Maldives OR "Sri Lanka” OR “first-generation” OR (MH "Immigrants+") OR (MM "Asia, Southern+") OR OR (MH " Transients and Migrants")  “industrialised countr*” OR “industrialised count*” OR “high-income countr*” OR “developed countr*” OR (MM "Developed Countries") OR Romania OR Panama OR Chile OR “Trinidad and Tobago” OR Uruguay OR Antigua and Barbuda OR Barbados OR Poland OR Hungary OR Croatia OR Oman OR Nauru OR Curacao OR “Saint Kitts” and Nevis OR “French Polynesia” OR Latvia OR Greece OR Slovakia OR Lithuania OR “Puerto Rica” OR “Turks and Caicos Islands” OR “Saudi Arabia” OR “Czech Republic” OR Bahrain OR Estonia OR Bahamas OR Slovenia OR Cyprus OR Aruba OR Brunei OR Malta OR “Sint Maarten” OR Greenland OR Kuwait OR “South Korea” OR Italy OR “New Caledonia” OR UAE OR Andorra OR Macau OR “San Marino” OR Israel OR Belgium OR “Hong Kong” OR Sweden OR “Cayman Islands” OR Qatar OR Singapore OR Iceland OR “Faroe Islands” OR Norway OR Luxembourg OR “Isle of Man” OR Ireland OR Bermunda OR USA OR UK OR Canada OR Switzerland OR Germany OR France OR Australia OR Netherlands OR “New Zealand” OR Finland OR Japan OR Denmark  factor* OR “social determinant*” OR (MM "Sociodemographic Factors") OR (MM "Social Factors") OR (MH "Life Style, Sedentary+")  Stress OR Depression OR Anxiety OR “acculturative stress” OR discrimination OR “racial discrimination” OR “social support” OR (MH " Anxiety Disorders+") |
| PsycINFO | (Migrant* OR Immigrant* OR Migration OR “CALD population*” OR “South Asia*” OR Afghanistan OR Pakistan OR Nepal OR Bangladesh OR Bhutan OR India OR Maldives OR “Sri Lanka” OR “first-generation” ) AND  (“industrialised countr*” OR “industrialized count* OR “high-income countr*” OR “developed countr*” OR Romania OR Panama OR Chile OR “Trinidad and Tobago” OR Uruguay OR “Antigua and Barbuda” OR Barbados OR Poland OR Hungary OR Croatia OR Oman OR Nauru OR Curacao OR “Saint Kitts” and Nevis OR “French Polynesia” OR Latvia OR Greece OR Slovakia OR Lithuania OR “Puerto Rica” OR “Turks and Caicos Islands” OR “Saudi Arabia” OR “Czech Republic” OR Bahrain OR Estonia OR Bahamas OR Slovenia OR Cyprus OR Aruba OR Brunei OR Malta OR “Sint Maarten” OR Greenland OR Kuwait OR “South Korea” OR Italy OR “New Caledonia” OR UAE OR Andorra OR Macau OR “San Marino” OR Israel OR Belgium OR “Hong Kong” OR Sweden OR “Cayman Islands” OR Qatar OR Singapore OR Iceland OR “Faroe Islands” OR Norway OR Luxembourg OR “Isle of Man” Ireland OR Bermunda OR USA OR UK OR Canada OR Switzerland OR Germany OR France OR Australia OR Netherlands OR “New Zealand” OR Finland OR Japan OR Denmark) AND  (factor* OR “social determinant*”) AND  (Stress OR Depression OR Anxiety OR “acculturative stress” OR discrimination OR “racial discrimination” OR “social support”) |
